# Supplementary material for: Enhanced Photoluminescence of Europium-Doped TiO2 Nanoparticles Using a Single-Source Precursor Strategy
Source: Molecules. 2024 Dec 10;29(24):5824. doi: 10.3390/molecules29245824 (PMC11678432; doi:10.3390/molecules29245824)
Supplement: Supplementary file 1 [file molecules-29-05824-s001.zip › molecules-3324633-supplementary.pdf]

molecules

Enhanced Photoluminescence of Europium-Doped TiO_2 Nanoparticles using a Single-Source Precursor Strategy

Violaine Mendez [1], Marlène Fabre [1], Thibaut Cornier [1], Françoise Bosselet [1], Stéphane Loridant [1], Sarah Asaad [2] and Stéphane Daniele [2,*]

[1] IRCELYON, CNRS, University Claude Bernard Lyon 1, UMR 5256, F-69100 Villeurbanne, France; violaine.mendez@gmail.com (V.M.); marlene.fabre@ircelyon.univ-lyon1.fr (M.F.); thibaut.cornier@ircelyon.univ-lyon1.fr (T.C.); francoise.bosselet@ircelyon.univ-lyon1.fr (F.B.); stephane.loridant@ircelyon.univ-lyon1.fr (S.L.)

[2] CP2M-ESCPE Lyon, CNRS, University Claude Bernard Lyon 1, UMR 5128, 43 Bd du 11 Nov. 1918, CEDEX, 69616 Villeurbanne, France; sara.asad@univ-lyon1.fr

* Correspondence: stephane.daniele@univ-lyon1.fr

Supplementary Materials

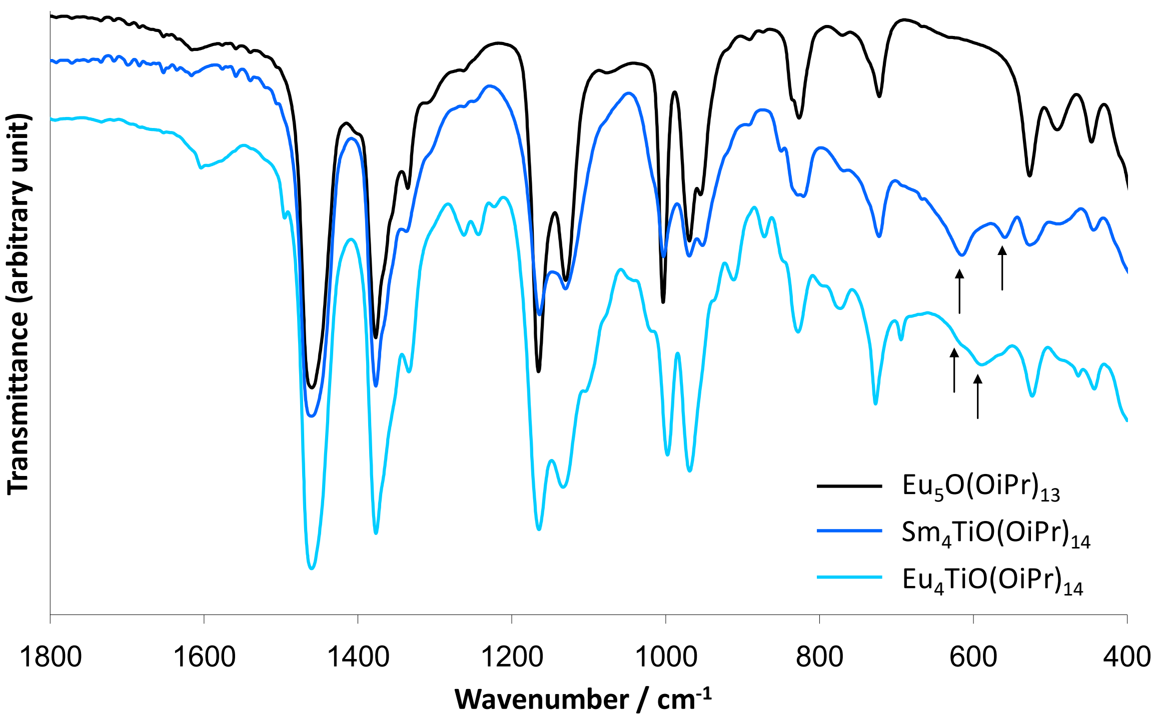

Figure S1. FT-IR spectra of $Eu_4Ti(OiPr)_{14}$ precursor compared to $Sm_4Ti(OiPr)_{14}$ and $Eu_5O(OiPr)_{13}$.

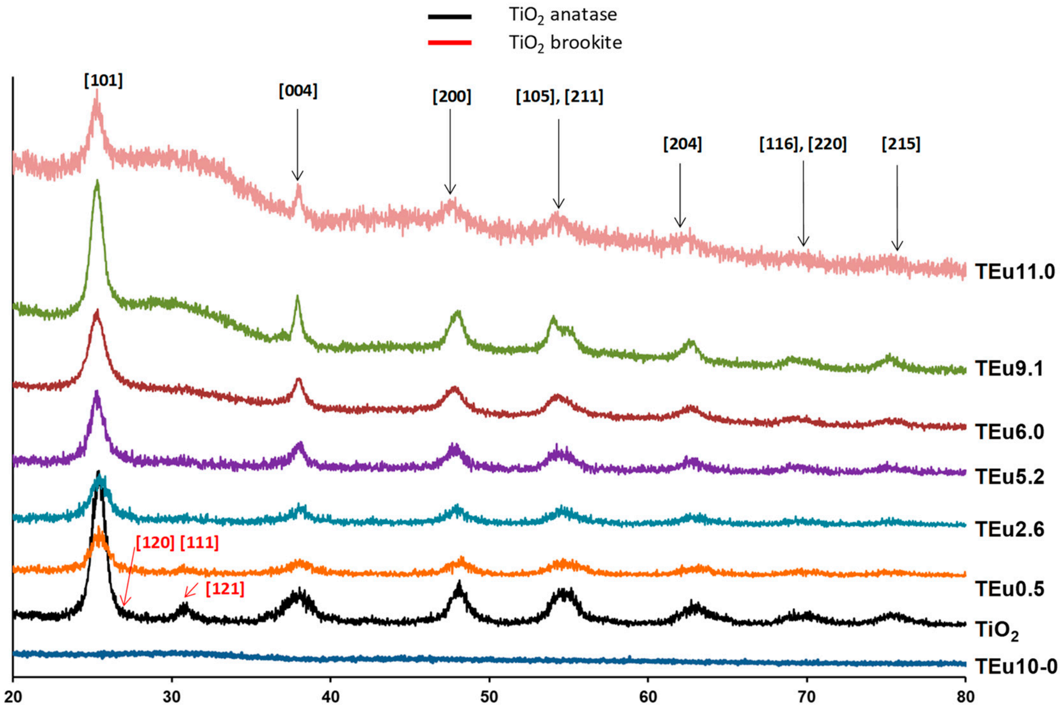

Figure S2. XRD patterns of as-prepared sol-gel samples.

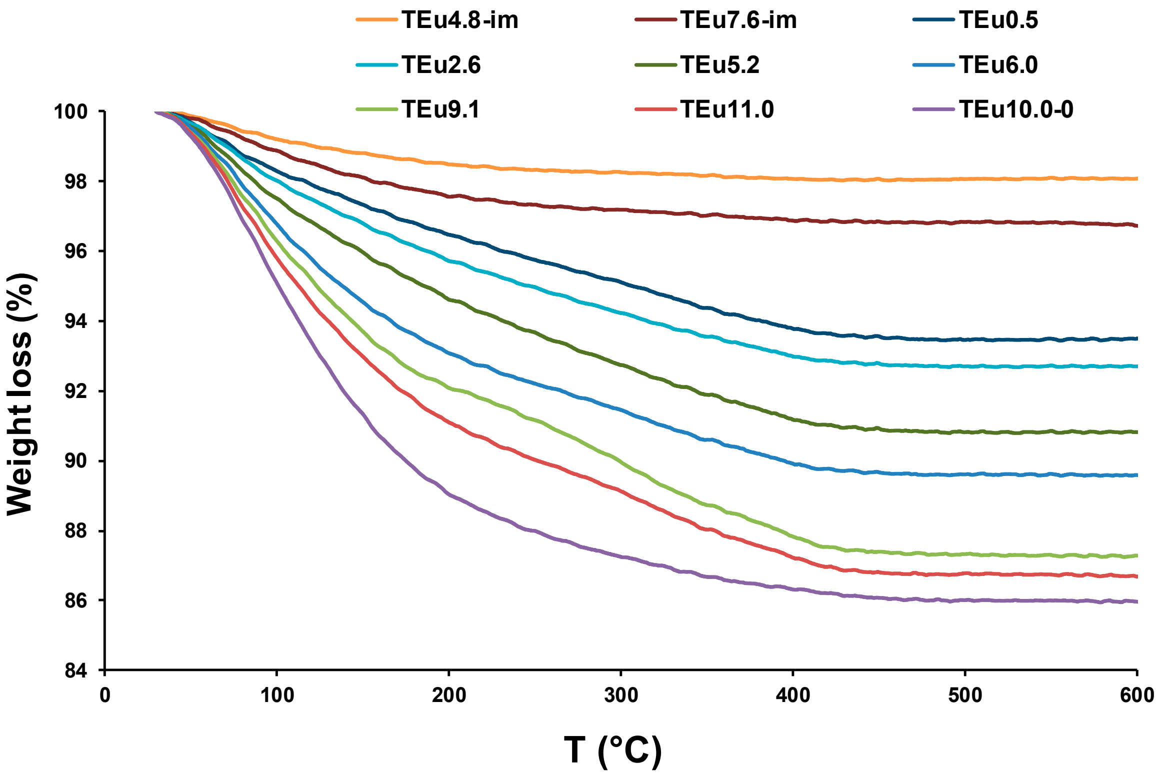

Figure S3. TGA patterns of as-prepared TEuX and TEuX-im samples.

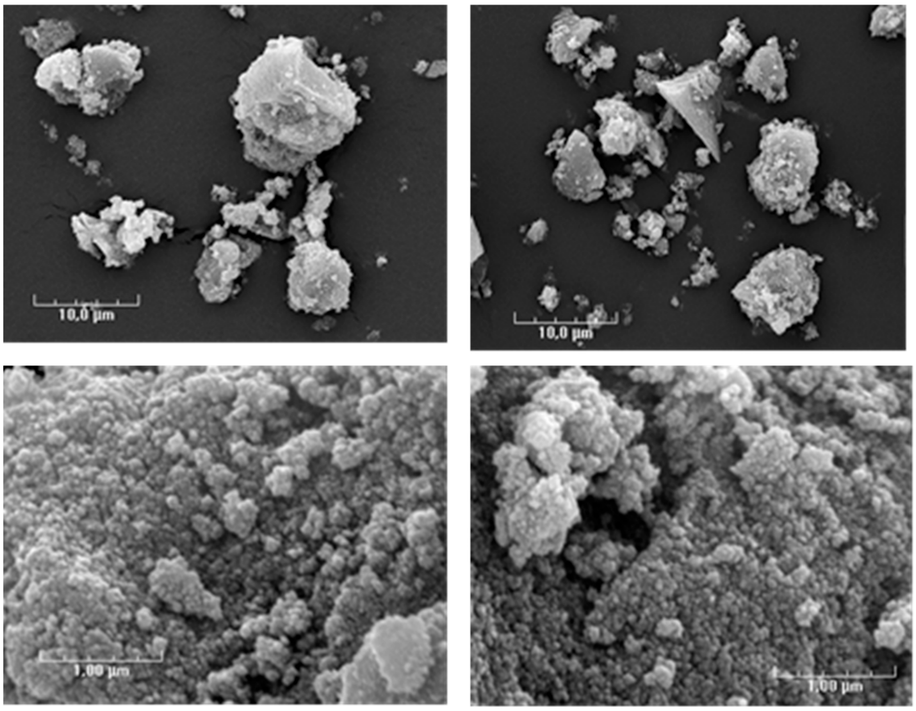

Figure S4. SEM images of TEu0.5 and TEu11.0 samples.

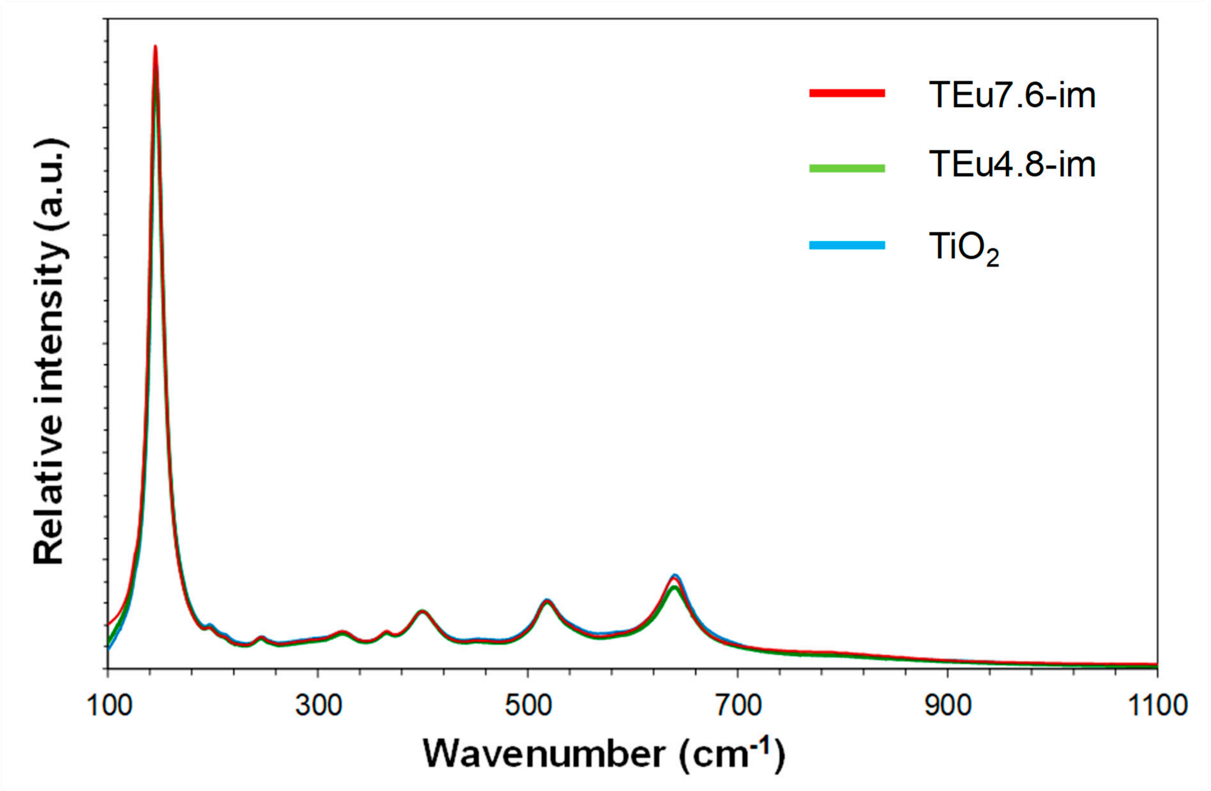

Figure S5. Raman spectra of TiO_2 and TEuX-im samples. The band at 147 cm^{-1} was used for normalization.

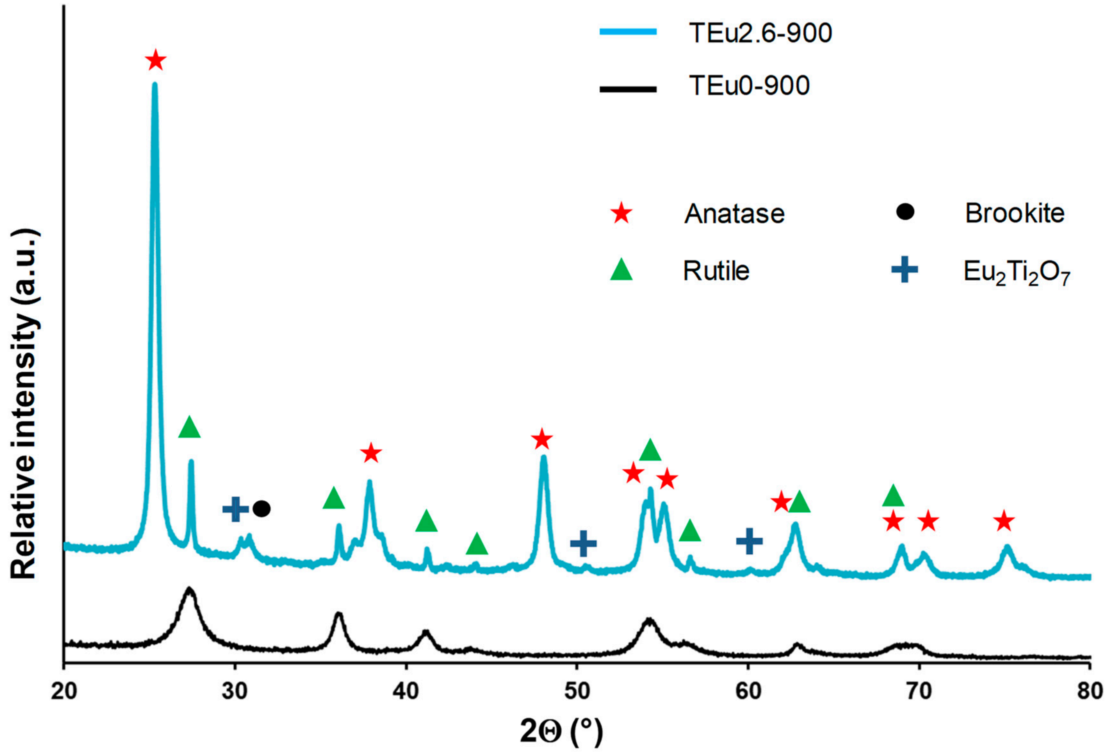

Figure S6. XRD patterns of TEu0-900 and TEu2.6-900 samples.

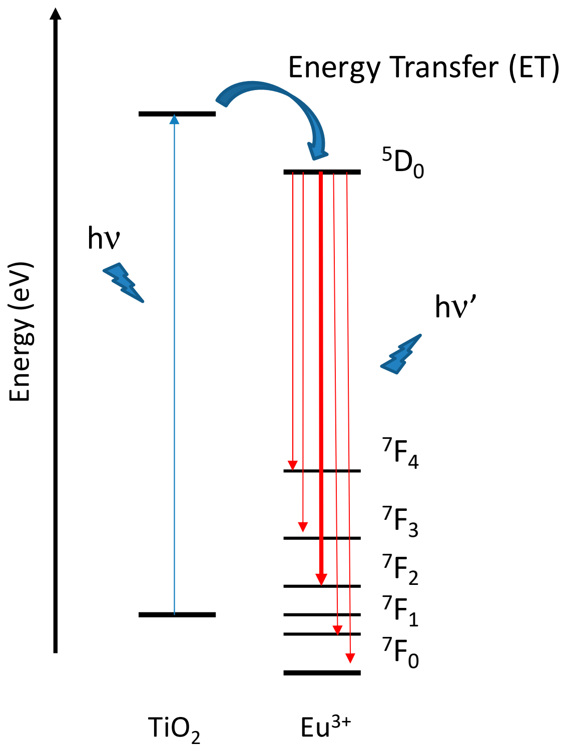

Figure S7: Jablonski diagram showing the excitation process of TiO_2 and energy transfer (ET) to Eu^{3+} levels ions and 4f emissions

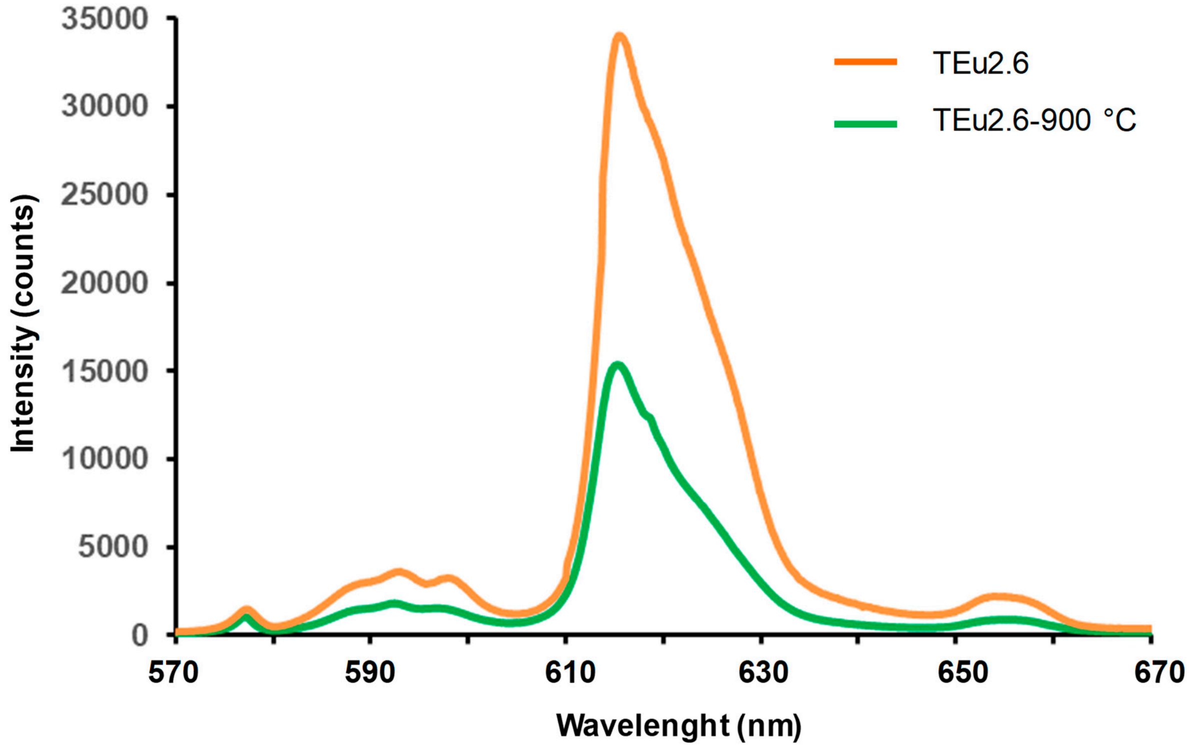

Figure S8: Photoluminescence spectra between 570 and 670 nm of TEu2.6 and TEu2.6-900 samples.

Table S1. Elemental analysis data (duplicate) of the TEuX and TEuX-im samples.

**Wavelength (nm)**	**412.97 - 381.697 - 393.048**
**Sample**	**Eu content (wt %)**
TEu0.5	1.03-1.07
TEu2.6	4.87-4.90
TEu5.2	9.34-9.54
TEu6.0	10.78-10.78
TEu9.1	15.93-15.97
TEu11.0	19.05-19.20
TEu2.7-im	4.82-5.23
TEu4.8-im	8.38-8.40
TEu7.6-im	13.39-13.47
TEu10.0-0	17.23-17.33

Table S2. Ti, O, C and Eu quantitative analysis XPS of the TEuX and TEuX-im samples.

**Quantitative analysis (at %)**				
**Sample**	**Ti**	**O**	**C**	**$Eu3d_{5/2}$**
**TEu10.0-0**	15.7	47.1	19.7	14.3
**TEu9.1**	18.0	49.6	15.3	15.0
**TEu4.8-im**	18.9	49.3	13.3	16.2
**Sample**	**Ti**	**O**	**C**	**$Eu4d_{5/2}$**
**TEu10.0-0**	17.5	52.5	22.0	4.4
**TEu9.1**	20.2	55.8	17.2	4.4
**TEu4.8-im**	21.9	57.0	15.4	3.0

Table S3. O1s and C1s XPS data of the TEuX and TEuX-im samples.

**Decomposition of O1s**	**a**		**b**		**c**	
**Sample**	**BE (eV)**	**Peak %**	**BE (eV)**	**Peak %**	**BE (eV)**	**Peak %**
**TEu10.0-0**	529.9	**61.9**	531.1	**34.4**	532.7	**3.7**
**TEu9.1**	529.9	**72.8**	531.1	**23.8**	532.7	**3.5**
**TEu4.8-im**	529.7	**75.2**	530.9	**23.2**	532.8	**1.7**
**Decomposition of C1s**	**a**		**b**		**c**	
**Sample**	**BE (eV)**	**Peak %**	**BE (eV)**	**Peak %**	**BE (eV)**	**Peak %**
**TEu10.0-0**	284.9	**19.7**	286.1	**63.0**	288.9	**17.3**
**TEu9.1**	285.0	**24.4**	286.1	**57.8**	288.7	**17.8**
**TEu4.8-im**	284.9	**20.9**	286.1	**60.4**	288.8	**18.8**
